# Selectivity in Genetic Association with Sub-classified Migraine in Women

**DOI:** 10.1371/journal.pgen.1004366

**Published:** 2014-05-22

**Authors:** Daniel I. Chasman, Verneri Anttila, Julie E. Buring, Paul M. Ridker, Markus Schürks, Tobias Kurth, on behalf of the International Headache Genetics Consortium

**Affiliations:** 1 Division of Preventive Medicine, Brigham and Women's Hospital, Boston, Massachusetts, United States of America; 2 Harvard Medical School, Boston, Massachusetts, United States of America; 3 Analytical and Translational Genetics Unit, Department of Medicine, Massachusetts General Hospital, Boston, Massachusetts, United States of America; 4 Program in Medical and Population Genetics, Broad Institute of MIT and Harvard, Cambridge, Massachusetts, United States of America; 5 Institute for Molecular Medicine Finland (FIMM), University of Helsinki, Helsinki, Finland; 6 Department of Neurology, University Hospital Essen, Essen, Germany; 7 Inserm Research Center for Epidemiology and Biostatistics (U897) - Team Neuroepidemiology, Bordeaux, France; 8 University of Bordeaux, College of Health Sciences, Bordeaux, France; Georgia Institute of Technology, United States of America

## Abstract

Migraine can be sub-classified not only according to presence of migraine aura (MA) or absence of migraine aura (MO), but also by additional features accompanying migraine attacks, e.g. photophobia, phonophobia, nausea, etc. all of which are formally recognized by the International Classification of Headache Disorders. It remains unclear how aura status and the other migraine features may be related to underlying migraine pathophysiology. Recent genome-wide association studies (GWAS) have identified 12 independent loci at which single nucleotide polymorphisms (SNPs) are associated with migraine. Using a likelihood framework, we explored the selective association of these SNPs with migraine, sub-classified according to aura status and the other features in a large population-based cohort of women including 3,003 active migraineurs and 18,108 free of migraine. Five loci met stringent significance for association with migraine, among which four were selective for sub-classified migraine, including rs11172113 (*LRP1*) for MO. The number of loci associated with migraine increased to 11 at suggestive significance thresholds, including five additional selective associations for MO but none for MA. No two SNPs showed similar patterns of selective association with migraine characteristics. At one extreme, SNPs rs6790925 (near *TGFBR2*) and rs2274316 (*MEF2D*) were not associated with migraine overall, MA, or MO but were selective for migraine sub-classified by the presence of one or more of the additional migraine features. In contrast, SNP rs7577262 (*TRPM8*) was associated with migraine overall and showed little or no selectivity for any of the migraine characteristics. The results emphasize the multivalent nature of migraine pathophysiology and suggest that a complete understanding of the genetic influence on migraine may benefit from analyses that stratify migraine according to both aura status and the additional diagnostic features used for clinical characterization of migraine.

## Author Summary

Migraine is among the most common and debilitating neurological disorders. Diagnostic criteria for migraine recognize a variety of symptoms including a primary dichotomous classification for the presence or absence of aura, typically a visual disturbance phenomenon, as well as others such as sensitivity to light or sound, and nausea, etc. We explored whether any of 12 recently discovered genetic variants associated with common migraine might have selective association for migraine sub-classified by aura status or nine additional migraine features in a population of middle-aged women including 3,003 migraineurs and 18,180 non-migraineurs. Five of the 12 genetic variants met the most stringent significance criterion for association with migraine, among which four had selective association with sub-classified migraine, including one that was selective for migraine without aura. At suggestive significance, all of the remaining genetic variants were selective for sub-classifications of migraine although no two variants showed the same pattern of selectivity. The selectivity patterns suggest very different contributions to migraine pathophysiology among the 12 loci and their implicated genes. Further, the results suggest that future discovery efforts for new migraine susceptibility loci would benefit by considering associations with sub-classified migraine toward the ultimate goals of more specific diagnosis and personalized treatment.

## Introduction

Migraine is one of the most common and debilitating neurological disorders and its clinical presentation can be quite variable [1]. Even when the diagnosis of migraine meets consensus criteria and can in most cases be clearly distinguished from other types of headaches (e.g. tension-type headache), phenotypic heterogeneity in migraine persists [2], [3]. The most pronounced heterogeneity in migraine is the dichotomous sub-classification according to the presence (MA) or absence (MO) of aura, which most commonly manifests as a visual disturbance that generally precedes an attack of headache fulfilling the criteria for migraine. Other characteristics that may be used to sub-classify migraine are features of the migraine attack, including pulsatile pain character, unilateral pain, photophobia, phonophobia, attack duration, nausea, aggravation by physical activity, severity that inhibits daily activities, and finally the frequency of attacks. The International Classification of Headache Disorders (ICHD) acknowledges all these characteristics either as diagnostic criteria for migraines or to distinguish different forms of migraine [4]. Although our understanding of the migraine and aura pathophysiology has substantially improved [5], many details of migraine aura and the role of other migraine features remain unclear.

The heterogeneity of migraine characteristics raises both a challenge and opportunity for using genetics to understand migraine pathophysiology. While the power to detect genetic associations will be degraded by potential misclassification due to the heterogeneity of the clinical presentation, associations that are selective for migraine with certain characteristics may help reveal detailed biological causes of migraine, and anticipate the potential of gene-based migraine classification and treatment. Genetics is known to be an important determinant of migraine with heritability estimated in the range 30–60%; and the heritability for MA is estimated somewhat higher than for MO [6]–[10]. Recent reports have described a greater number of highly significant common genetic variants for MO than MA in genome-wide analyses, as well as only partial overlap between the sets of identified genes [11]–[13]. One possible explanation of the apparent discrepancy between heritability estimates and yield of genome-wide significant associations may be different genetic contributions to MA v. MO with, for example, the former possibly characterized by genetic variants that are rarer or more population specific, or more heterogeneous compared with the latter [14], [15]. Similarly, it is possible that a dichotomy in the genetic architecture may underlie the additional features that often accompany migraine headache, i.e. nausea, photophobia, etc.

Here we apply a likelihood-based analytic framework [16] to explore the possibility of preferential associations with sub-classified migraine in a population-based cohort of women for 12 single nucleotide polymorphisms (SNPs) arising in recent genome-wide association studies (GWAS) for migraine overall, MO, or MA [13]. Enforcing strict significance thresholds, we find that five SNPs are associated with migraine in our cohort among which four had selective association with sub-classified migraine. At suggestive significance, 11 loci were associated with migraine and all but one displayed selective association with sub-classified migraine. However, none of the patterns of selective association according to aura status or the other characteristics was shared by more than one SNP. The findings suggest that the recently reported genetic variants influence the underlying pathophysiology of migraine in very different ways.

## Results

Among the Women's Genome Health Study (WGHS) participants of European ancestry with available genetic data, there were 3,003 women who reported active migraine at baseline, defined as migraine experienced in the year prior to enrollment, compared with 18,108 who had never experienced migraine (Table 1). An additional 2,119 reported having experienced migraine previously but not in the preceding year and thus were not sub-classified according to migraine characteristics. These participants were excluded from the main analysis. Compared with non-migraineurs, active migraineurs tended to be younger, have higher BMI, be more likely to use hormone replacement therapy but less likely to smoke. Thirty nine percent (N = 1,177) of the WGHS participants with active migraine reported aura, compared with 61% (N = 1,826) who did not (Table 2). The prevalence of features associated with migraine ranged from 34% for pain aggravated by physical activity to 78% for duration of 4–72 hours.

**Table 1:** Demographic characteristics of the WGHS according to migraine status.

	active migraine#	no migraine#	p*
N	3,003	18,108	
Age (yrs)	51.5 (48.4–55.9)	53.3 (49.1–59.7)	<0.001
Height (inches)	65 (63.0–66.0)	65 (63.0–66.0)	0.45
BMI (kg/m^2^)	25.0 (22.6–28.4)	24.9 (22.5–28.3)	0.03
Ever smoking	1,375 (45.8%)	8,953 (49.5%)	2.0×10^−4^
BP (5 category)			0.14
SBP<120 mmHg, DBP<75	1,004 (33.7%)	6,041 (33.8%)	
SBP<130, DBP<85	989 (33.2%)	5,787 (32.4%)	
SBP<140, DBP<90	555 (18.7%)	3,340 (18.7%)	
SBP<160, DBP<95	352 (11.8%)	2,354 (13.2%)	
SBP> = 160, DBP> = 95	75 (2.5%)	365 (2.0%)	
Hormone replacement therapy use	1,434 (47.9%)	7,723 (42.7%)	<0.001
History of diabetes	55 (1.8%)	481 (2.7%)	0.01

# median (inter-quartile range) or N (%).

*p-value from t-test (continuous variables) or chi-square test (categorical variables).

**Table 2:** WGHS active migraineurs (N = 3,003*) with aura or migraine characteristic.

Migraine characteristic	yes – N (fr.)	no – N (fr.)
aura	1,177 (0.39)	1,826 (0.61)
pulsating pain	1,591 (0.53)	1,412 (0.47)
unilateral pain	1,791 (0.60)	1,212 (0.40)
phonophobia	1,232 (0.41)	1,771 (0.59)
photophobia	1,976 (0.66)	1,027 (0.34)
duration of 4–72 hours	2,348 (0.78)	655 (0.22)
nausea	1,958 (0.65)	1,045 (0.35)
pain aggravation by physical activity	1,017 (0.34)	1,986 (0.66)
inhibition of daily activities	1,499 (0.50)	1,504 (0.50)
migraine attack frequency ≥6/year	1,050 (0.35)	1,953 (0.65)

*An additional 2,119 WGHS participants reported prior but not active migraine compared with 18,172 WGHS participants reported never experiencing migraine.

Applied to 12 SNPs (Table S1) recently reported with genome-wide association [13], the statistical model selection procedure with the Bayesian information criterion (BIC) penalty (see Methods for model selection approach) identified six SNPs with evidence for migraine association in the WGHS displaying selectivity according to one or more of the characteristics accompanying a migraine attack (Table 3). To estimate the significance of the models selected for each combination of SNP and migraine sub-classification, an empirical approach was used to control for multiple hypothesis testing (see Methods). In particular, permutation of genotype assignments to individuals was used to estimate: 1) for each combination of SNP and migraine sub-classification, the probability of selecting a “non-null” model among six models tested with the BIC (Table S2A), 2) for each combination of SNP and migraine sub-classification, an empirical p-value for the analytic log-likelihood (LLR) test of the BIC selected model (Table S2B), and 3) an overall p-value for each SNP correcting the empirical p-value in 2) for model selection across all 10 migraine sub-classifications (Table S2C). The empirical p-values selecting a non-null model for each combination of SNP and migraine subtype were in the range 0.0002–0.0066. For 2), the six SNPs with “non-null” BIC-selected model for at least one migraine sub-classification had LLR tests with a maximum empirical p-value 0.0032 before correction for multiple testing, among which the five excluding rs13208321 (*FHL5*) were significant after correcting for testing across the 10 migraine sub-classifications (“*” symbol, Table 3). SNP rs7577262 (*TRPM8*) was significantly associated with migraine, correcting for testing across all 10 sub-classifications, but displayed no selectivity for any of the migraine-associated characteristics. The remaining four significant SNPs were selective for one or more migraine associated characteristics. For example, SNP rs1172113 (*LRP1*) was preferentially associated with the migraine without aura, i.e. MO (“inverse subset”), and also for migraine with duration 4–72 hours (“subset”). Models distinguishing status of the other characteristics except pulsatile pain were selected for at least one of the SNPs meeting significance thresholds for multiple testing. However, none of the characteristics showed selective association shared by all SNPs.

**Table 3:** Inheritance model# selected with the BIC.

			migraine characteristic∧									
SNP	chr:pos (b. 37)	genomic context	aura	pulsation	unipain	sound	light	longdur	nausea	aggrphys	inhibit	freq
rs2651899	1:3083711	*PRDM16*	basic*	basic*	basic*	basic*	sub.*	basic*	sub.*	sub.*	sub.*	basic*
rs10915437	1:4183005	near *AJAP1*	-	-	-	-	-	-	-	-	-	-
rs12134493	1:115677945	near *TSPAN2*	basic*	basic*	sub.*	sub.*	sub.*	basic*	basic*	basic*	basic*	sub.*
rs2274316	1:156446241	*MEF2D*	-	-	-	-	-	-	-	-	-	-
rs7577262	2:234818868	*TRPM8*	basic*	basic*	basic*	basic*	basic*	basic*	basic*	basic*	basic*	basic*
rs6790925	3:30480084	near *TGFBR2*	-	-	-	-	-	-	-	-	-	-
rs9349379	6:12903956	*PHACTR1*	-	-	-	-	-	-	-	-	-	-
rs13208321	6:96860353	*FHL5*	-	-	-	-	-	-	sub.	-	-	-
rs4379368	7:40466199	*C7orf10*	-	-	-	-	-	-	-	-	-	-
rs10504861	8:89547931	near *MMP16*	-	sub.	sub.*	sub.*	sub.*	sub.*	sub.*	-	-	-
rs6478241	9:119252628	*ASTN2*	-	-	-	-	-	-	-	-	-	-
rs11172113	12:57527282	*LRP1*	inv. sub.*	basic*	basic*	basic*	basic*	sub.*	basic*	basic*	basic*	basic*

# Models (see Methods): “-” = null, basic, “sub” = subset, “inv. sub” = inverse subset.

∧ Migraine characteristics: aura, pulsation, unipain ( = unilateral pain), sound ( = phonophobia), light ( = photophobia), duration of 4–72 hours ( = longdur), nausea, aggravation by physical activity ( = aggrphys), severity inhibits daily activities ( = inhibit), ≥6 attacks/year ( = freq).

“*” indicates models with significant LLR test p-values (<0.05) after adjustment for multiple hypothesis testing (see Methods).

Model selection with the Akiake information criterion (AIC) penalty was less stringent, identifying selective associations according to migraine characteristics for all SNPs except rs7577262 (*TRPM8*) (Table 4). The greater number of non-null models could be explained by the more permissive model selection found in permutation analysis that estimated the fraction of non-null models by chance in the range 0.16–0.35 (Table S3A). Nevertheless, all SNPs except rs10915437 (near *AJAP1*) had at least one model with nominally significant empirical p-value for the LLR test (i.e. <0.05), and the same five SNPs that had empirically significant LLR tests in the BIC model selection were also significant in the AIC model selection, although in some cases different models were selected (Tables 4 & S3 A, B, C). Thus, for SNP rs10504861 (near *MMP16*), the AIC selected the “inverse subset” model for aura (i.e. MO) and a “basic” model for migraine characterized by aggravation by physical activity, inhibition of daily activities, or attack frequency ≥6/year compared with the BIC selected “null” model for these characteristics. Similarly, SNP rs13208321 (*FHL5*) was identified as “null” with BIC model selection but as “inverse subset” for aura by the AIC as well as “subset” for other features. Additional differences at the five SNPs included selection of “general” rather than “subset” models for phonophobia and migraine attack frequency ≥6/year at rs12134493 (*TSPAN*), and “general” rather than “basic” or “subset” models respectively for phonophobia and aggravation by physical activity at rs2651899 (*PRDM16*). Some of the remaining SNPs had AIC-selected models with nominally significant empirical LLR p-values (Table S3B), although none of these models was significant after correction for multiple testing (Table S3C). Nevertheless, the nominally significant selective models highlighted additional differences compared with the BIC penalized analysis, among which “inverse subset” models for aura (i.e. MO) were selected at rs9349379 (*PHACTR1*) and rs6478241 (*ASTN2*).

**Table 4:** Inheritance model# selected with the AIC.

			migraine characteristic∧									
SNP	chr:pos (b. 37)	genomic context	aura	pulsation	unipain	sound	light	longdur	nausea	aggrphys	inhibit	freq
rs2651899	1:3083711	*PRDM16*	basic*	basic*	basic*	general*	sub.*	basic*	sub.*	general*	sub.*	basic*
rs10915437	1:4183005	near *AJAP1*	inv. sub.	sub.	-	-	-	-	-	-	-	-
rs12134493	1:115677945	near *TSPAN2*	basic*	basic*	sub.*	general*	sub.*	basic*	basic*	basic*	basic*	general*
rs2274316	1:156446241	*MEF2D*	-	general	-	-	-	-	-	-	sub.	sub.
rs7577262	2:234818868	*TRPM8*	basic*	basic*	basic*	basic*	basic*	basic*	basic*	basic*	basic*	basic*
rs6790925	3:30480084	near *TGFBR2*	-	-	sub.	sub.	general	sub.	general	sub.	sub.	sub.
rs9349379	6:12903956	*PHACTR1*	inv. sub.	basic	basic	basic	basic	basic	basic	inv. sub.	basic	Basic
rs13208321	6:96860353	*FHL5*	inv. sub.	basic	basic	basic	basic	sub.	sub.	sub.	sub.	Basic
rs4379368	7:40466199	*C7orf10*	basic	sub.	basic	basic	basic	basic	basic	sub.	sub.	sub.
rs10504861	8:89547931	near *MMP16*	inv. sub.	sub.	sub.*	sub.*	sub.*	sub.*	sub.*	basic	basic	basic
rs6478241	9:119252628	*ASTN2*	inv. sub.	basic	sub.	basic	sub.	basic	Basic	basic	sub.	basic
rs11172113	12:57527282	*LRP1*	inv. sub.*	basic*	basic*	basic*	basic*	sub.*	basic*	basic*	basic*	basic*

# Models (see Methods): “-” = null, basic, “sub” = subset, “inv. sub” = inverse subset, general.

∧ Migraine characteristics: aura, pulsation, unipain ( = unilateral pain), sound ( = phonophobia), light ( = photophobia), duration of 4–72 hours ( = longdur), nausea, aggravation by physical activity ( = aggrphys), severity inhibits daily activities ( = inhibit), ≥6 attacks/year ( = freq).

“*” indicates models with significant LLR test p-values (<0.05) after adjustment for multiple hypothesis testing (see Methods).

Using the same BIC and AIC model selection methodology, there were few differences in the SNP associations between the 3,003 active migraineurs and the 2,119 former migraineurs who were excluded from the current analysis due to lack of information related to migraine sub-classification (Table 5). With the BIC penalty, four SNPs were assigned “non-null” models, all of which were of the “basic” type, implying no statistical difference in SNP association between active and former migraine status. With the AIC penalty, five additional SNPs were assigned “non-null” models and only one, rs10504861 (near *MMP16*), displayed preferential association suggesting stronger association with active migraine.

**Table 5:** Testing selective association for active (N = 3,003) compared with former migraineurs (N = 2,119).

			model selected*	
SNP	chr:pos (b. 37)	genomic context	BIC	AIC
rs2651899	1:3083711	*PRDM16*	basic	basic
rs10915437	1:4183005	near *AJAP1*	-	-
rs12134493	1:115677945	near *TSPAN2*	basic	basic
rs2274316	1:156446241	*MEF2D*	-	-
rs7577262	2:234818868	*TRPM8*	basic	basic
rs6790925	3:30480084	near *TGFBR2*	-	-
rs9349379	6:12903956	*PHACTR1*	-	basic
rs13208321	6:96860353	*FHL5*	-.	basic
rs4379368	7:40466199	*C7orf10*	-	basic
rs10504861	8:89547931	near *MMP16*	-	sub.
rs6478241	9:119252628	*ASTN2*	-	basic
rs11172113	12:57527282	*LRP1*	basic	basic

*model definitions as in Tables 3 and 4.

**Figure 1:**
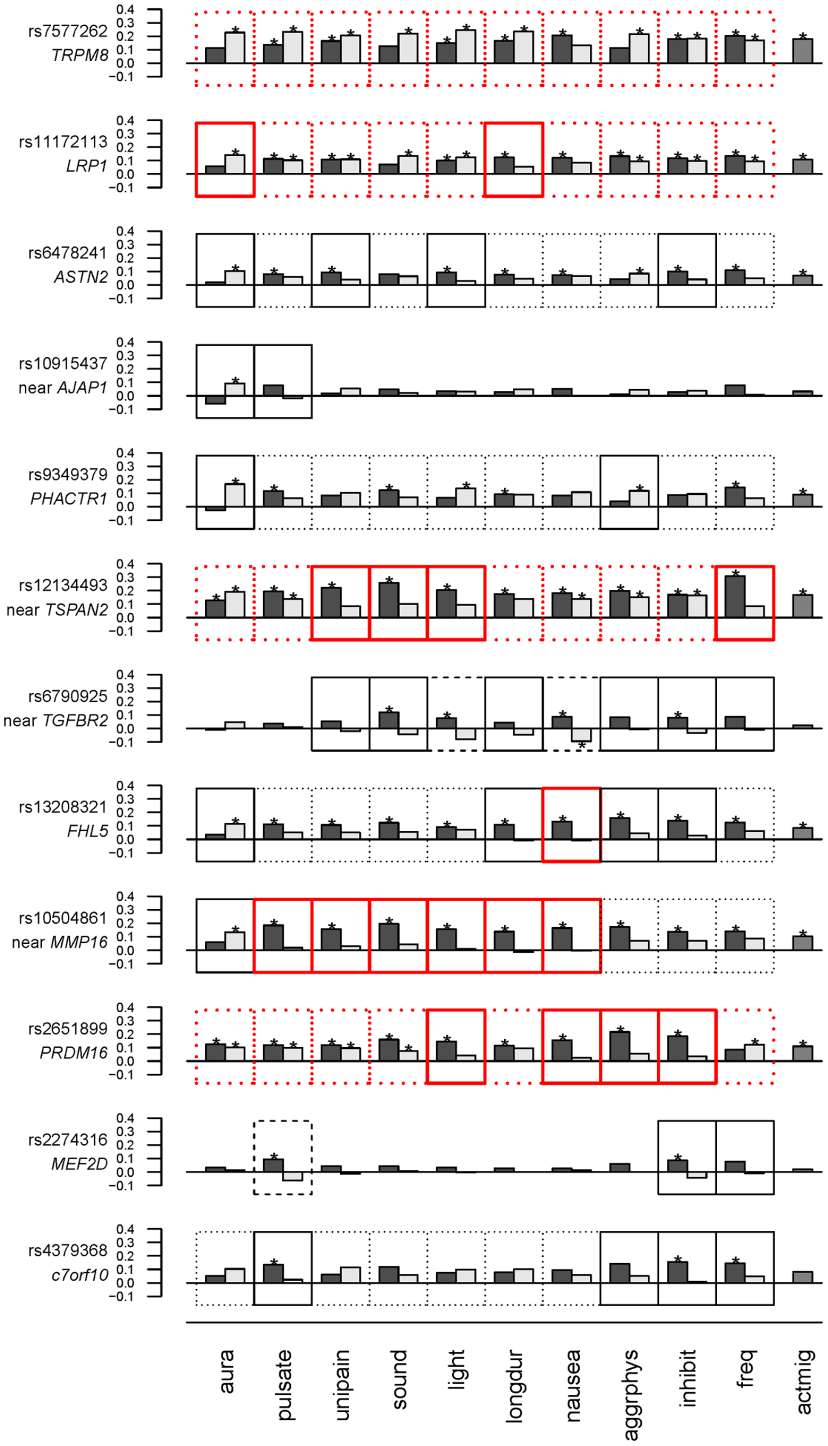
Estimates (beta coefficients) for association in the WGHS from logistic models for each of the 12 candidate SNPs as predictors of migraine accompanied by aura or other characteristics (black bars), or not (gray bars). Significant associations are indicated with “*” (see also Table S4). Model selection results (Tables 3 & 4) are indicated with outlines around each plot as follows: Non-null models selected using the BIC are indicated with a heavy red outline, while non-null models selected using the AIC are indicated with a thin black outline. “Subset” or “inverse subset” models (see Methods) are indicated with a solid outline, “basic” models are indicated with a dotted outline, and “general” models are indicated with a dashed outline. Migraine characteristics considered were aura, pulsating pain ( = pulsate), unilateral pain ( = unipain), phonophobia ( = sound), photophobia ( = light), duration of 4–72 hours ( = longdur), nausea, aggravation by physical activity ( = aggrphys), inhibition of daily activities ( = inhibit), ≥6 attacks/year ( = freq). The rightmost column (actmig) indicates association estimates for active migraineurs, irrespective of status for the characteristics (see Methods). The order of SNPs is derived from clustering as in Figure S1. See also Methods.

To examine the model selection results in more detail, the association effects of each SNP for migraine sub-classified according to presence or absence of each characteristic were estimated by logistic regression (Table S4) and depicted in Figure 1. To aid in presentation of the results, SNPs were ordered according to clustering based on the normalized differences in the association effects for migraine accompanied with or without the characteristics (Fig. S1). The clustering thus juxtaposed SNPs with approximately similar patterns of selectivity across aura status and the other migraine-associated characteristics. At the top of Figure 1, SNPs rs7577262 (*TRPM8*), rs11172113 (*LRP1*), rs6478241 (*ASTN2*), rs10915437 (near *AJAP1*), and rs9349379 (*PHACTR1*) form a cluster with relatively less pronounced differences in association by stratum status of the migraine-associated characteristics. In particular, associations with SNP rs7577262 (*TRPM8*) displayed associations essentially undifferentiated by stratum status, as reflected also by exclusively BIC-selected “basic” models (Table 2 and Fig. 1, boxes with heavy dotted outline). SNP rs11172113 (*LRP1*) in this group had mostly undifferentiated associations, except for stratum-specific associations according to aura status (for MO, beta [SE] = 0.14 [0.036], p = 8.3×10^5^ compared with MA, beta [SE] = 0.057 [0.043], p = 0.19) and migraine duration 4–72 hours (beta [SE] = 0.12 [0.032], p = 0.00012) but not duration under four hours (beta [SE] = 0.054 [0.058], p = 0.34), as reflected also by “inverse subset” and “subset” models with the BIC (boxes with heavy solid outline in Fig. 1), respectively. In the middle of Figure 1, SNPs rs10504861 (near *MMP16*), rs13208321 (*FHL5*), rs2651899 (*PRDM16*), and rs12134493 (near *TSPAN2*) all show significant associations for migraine sub-classified according to one or more of the migraine characteristics. These findings are consistent with corresponding “subset” and or “inverse subset” models from the BIC (Table 4). The remaining three SNPs have a mixture of stratum independent and stratum specific associations that differentiate them from the other two clusters. Throughout the array additional differences in the association effects according to stratum status are observed for many SNPs as again reflected also in the “subset” or “inverse-subset” models from the AIC model selection (Table 5), for example the differences according to aura status at rs9349379 (*PHACTR1*) and rs6478241 (*ASTN2*) as above rs10915437 (near *AJAP1*), and rs13208321 (*FHL5*).

Only two SNPs, rs6790925 (near *TGFBR2*) and rs2274316 (*MEF2D*), were found to have “null” models for stratification according to aura status, and inspection of the beta coefficients (Fig. 1, Table S4) suggested additional qualitative differences from the other SNPs. In spite of the very small effects on MA and MO, and essentially no association with active migraine overall, both SNPs had appreciable and significant associations for migraine accompanied by one or more of the characteristics, even showing significant protective association for rs6790925 and migraine without nausea (beta [SE] = −0.095 [0.048], p = 0.047) and without photophobia (beta[SE] = −0.044[0.037], p = 0.23), or for rs2274316 and migraine without pulsation (beta[SE] = −0.062[0.042], p = 0.14), although only the first of these combinations was significant. These patterns of association are consistent with the “general” models (dashed line, Fig. 1) that were selected with the AIC penalty and are characterized by different SNP allele frequencies in all three sub-groups, i.e. unaffected individuals as well as migraineurs either accompanied or not with the characteristics.

## Discussion

Examining the 12 SNPs recently discovered for association with migraine, we demonstrated significant preferential associations with MO compared to MA at high stringency for one SNP (rs11172113, *LRP1*) and at lower stringency for five SNPs. Of these, only rs10504861 (near *MMP16*), had been discovered initially in an association analysis specifically targeting MO. Four additional SNPs had no evidence of selectivity for aura status in their associations with migraine. SNP rs7577262 (*TRPM8*) in particular was highly significant for association with active migraine but not selective for aura or any of the other characteristics. It is perhaps relevant that *TRPM8*, the candidate gene for this SNP, is thought to mediate the sensation of pain rather than specific neurological or vascular functions that might more directly differentiate the pathophysiology of the migraine sub-classes [17]. The final two SNPs were not associated with active migraine, MA, or MO but were associated with migraine accompanied by one or more of the other migraine-specific characteristics, implying that these characteristics may be more relevant to the underlying pathophysiologic consequences of these genetic variants than aura status. Among the candidate functions for loci other than *TRPM8*, *PRDM16* has roles in cardiac development [18] and directing developmental cell fates toward brown fat or skeletal muscle [19], SNPs near the matrix metalloproteinase gene *MMP16* have been associated at genome-wide significance with psychiatric conditions [20] and non-syndromic cleft lip [21], and *LRP1*, encoding the LDL receptor-related protein 1 with molecular functions in endocytosis in several settings, has been implicated by GWAS in lipid homeostasis [22], lung function [23], abdominal aortic aneurysm [24], and transport of beta-amyloid in the brain [25]. The function of *TSPAN2*, the final candidate gene with BIC-selective association in the WGHS, is largely unknown, but it belongs to the tetraspanin family that has been linked to signal transduction [26]. Thus, the genetic architecture of migraine appears to reflect a multivalent pathophysiology and, from the dual perspectives of statistical power and understanding biology, association strategies that rely on the conventional dichotomy according to aura status may not be the sole or even best approach for genetic dissection of migraine.

Instead, the patterns of selective association with migraine accompanied by the non-aura characteristics may be at least as important to understanding migraine pathophysiology as the selectivity toward aura. All of the SNPs (except rs7577262) displayed some selectivity according to the non-aura characteristics that commonly accompany migraine, at least with the AIC penalty, and there was at least one SNP with a selective association for each characteristic even with the BIC penalty, which enforced a very high stringency in model selection. No pair of SNPs shared an identical patter of subtype associations. The selective associations are likely not to reflect, trivially, the contribution of the WGHS population to discovery of the candidate SNPs since sub-classification of migraine was not used in the original discovery. Moreover, the WGHS contribution to the main discovery analysis in the previous study [13] included a total of 5,122 migraineurs, as the combination of the 3,003 active migraineurs analyzed here and an additional 2,119 WGHS participants who reported having had migraine in their life but not in the year prior to enrollment. Thus, the migraineurs in the present analysis are a subset of those used in the published meta-analysis, even as the WGHS contributed approximately one-fifth of the total cases in that study. Only rs10504861 (near *MMP16*) in the previous analysis was identified exclusively in a previous sub-analysis restricted to MO and therefore including only the 1,826 WGHS MO cases rather than the 5,122 cases with history of any migraine. In contrast, rs10915437 (near *AJAP1*) and rs6790925 (near *TGFBR2*) were discovered exclusively among clinic-based samples, excluding the WGHS altogether. However, rs11172113 (*LRP1*) rather than rs10504861 was identified as an MO-specific in the analysis using the BIC penalized model selection. Thus, the design of the previous discovery meta-analysis is expected to confer only minimal bias at most in the selectivity of associations presented here, especially selective associations identified with the BIC penalty and those identified for sub-classifications other than the MA v. MO dichotomy.

There has been a suggestion that migraineurs who remit, i.e. appear to no longer experience migraine, may have different underlying pathophysiology from those who do not. This difference may have a partial genetic basis that might extend to differences in the selective associations reported here. Our data do not allow exploration of this possibility directly since migraineurs who did not report active migraine in the WGHS at baseline were not sub-classified according to aura status or the other migraine characteristics. However, we note that model selection for SNP associations with active compared with former migraine status suggested that the associations with overall migraine in the two groups were largely similar. The exception was rs10504861 (near *MMP16*) that displayed selective associations using the stringent BIC (Table 3) and also a preferential association with active compared with former migraineurs (Table 5).

One potential, though ultimately not robustly supported, explanation of the subtype associations might be that they simply reflect associations among individuals suffering a greater severity of migraine rather than selectivity for specific features. If this were the case, then one would expect that selectivity patterns might be highly correlated, perhaps especially with associations according to strata for migraine attack frequency, one measure of migraine severity. However, these patterns were not observed. First, the patterns of selective associations are not shared by any of the SNPs. Second, some SNPs show no selectivity (i.e. “basic” model) for migraine attack frequency ≥6/year but selectivity (i.e. “subset” or “inverse subset” models) for other characteristics, for example aura status (rs11172113 [*LRP1*], rs6478241 [*ASTN2*], rs13208321 [*FLH5*], rs10504861 [near *MMP16*]). Finally, among SNPs where there is a selective association with migraine characterized by attack frequency ≥6/year, there are few similarities among the associations with sub-classification based on the other characteristics. For example, rs12134493 (near *TSPAN2*), which is highly selective for migraine with attack frequency ≥6/year, also shows selectivity for unilateral pain, phonophobia, and photophobia, but not for pulsation, duration of 4–72 hours, aggravation by physical activity, and inhibition of daily activities, all features that show selective association with other SNPs, including SNPs that are also selective for high frequency migraine.

Several strengths and limitations should be considered when interpreting our results. Strengths include the large, homogeneous population-based sample of middle-aged women of European ancestry who were apparently healthy at study entry, as enforced specifically by a lack of overt CVD or cancer at baseline. Thus, the WGHS is very well-powered for the migraine sub-classification analysis presented here and further represents an age range in which migraine is relatively prevalent. Limitations include the self-reported nature of migraine and sub-phenotypes, which may result in misclassification. Other, comparably ascertained and well-powered cohorts that also include ascertainment of migraine sub-phenotypes and genotype information are not readily available and this circumstance limited our ability to replicate the analysis. Instead, we used a permutation procedure to establish significant thresholds consistent with multiple hypothesis testing. The study also does not address genetic associations with sub-classified migraine in other groups including women younger than 45, men, or children, nor does it address explicitly the genetic underpinnings of sub-classification in migraine with a strong familial inheritance pattern. Further targeted studies are warranted to address these issues.

The selective genetic associations with sub-classified migraine provide a glimpse into the future possibility of resolving some of the heterogeneity in migraine. Sub-classification of migraineurs according to combinations of migraine-associated characteristics potentially representing more clinically homogeneous sub-groups has been suggested as one approach [2], [27]. However, sub-groups of migraineurs cannot be unambiguously defined based on discrete patterns of co-occurrence of migraine-associated characteristics. Because of this ambiguity, applying the present statistical methodology to test for selective genetic associations with such sub-groups is much more complex than analyses based on individual migraine characteristics. In considering alternative approaches to solving the complex presentation and pathophysiology of migraine, ongoing research experience in the genetics of psychiatric disorders may be relevant. Psychiatric disorders are notoriously difficult to diagnose, a challenge that also extends to devising optimal treatment. Attempts to classify psychiatric disorders on the basis of clinical symptoms alone, as for example by the updated diagnosis criteria in the recently published DSM-5, are controversial [28], [29]. At the same time recent genome-wide genetic analyses have revealed both different and shared causal genetic loci across multiple psychiatric disorders with distinct diagnoses on the basis of clinical presentation alone [30], [31]. It is not hard to imagine that increasingly detailed clinical and genetic characterization may ultimately coalesce into integrated and more reliable diagnostic criteria for these psychiatric conditions. Such combined clinical and genetic strategies for improved classification may be imagined also for migraine, although they would likely require establishment of a larger number of genetic loci than the 12 robust loci explored in the current analysis.

Nevertheless, improved classification of migraine may help identify the most important pathophysiological pathway(s) in a given migraine patient and may allow for prioritization of treatment options. In this respect, discovery of more loci and therefore genes relevant to migraine in future genome-wide studies may provide further understanding of the complete set of biological interactions that underlie migraine in its various forms. Knowledge of these interactions may guide development of novel therapeutic strategies. The same knowledge may also be translated toward the ultimate clinical goal of delivering the most individually targeted therapy in treating migraine.

## Methods

### Study population

#### The Women's Genome Health Study (WGHS)

The WGHS [32] is a large population-based cohort for genetic analysis and includes individuals who provided a blood sample at baseline in the Women's Health Study (WHS) [33], [34], a randomized, placebo controlled trial of aspirin and vitamin E in primary prevention of cardiovascular disease and cancer among apparently healthy female healthcare professionals, aged 45 years or older at baseline in 1992–1995. Migraine in the WHS was ascertained at baseline by self-report as described previously [35], [36]. Briefly, participants were asked at baseline: “Have you ever had migraine headaches?” and “In the past year, have you had migraine headaches?” Responses to these questions were used to classify participants with no history of migraine, “active” migraine, i.e. migraine experienced within the past year, or “prior” migraine, i.e. migraine experienced more previous to the past year. Participants reporting active migraine were further queried for detailed characteristics of their migraine attacks including: the presence of aura status or premonition of an attack, the frequency of attacks (e.g. daily, weekly, etc.), the duration of attacks (4–72 hours), whether attacks were accompanied by nausea or sensitivity to light or sound, whether the pain had a unilateral location or a pulsating quality, and whether the pain was aggravated by physical activity or inhibited daily activity. Responses to these questions allowed classifications of migraineurs according to modified ICHD-2 criteria. In a subset of 1,675 participants from the Women's Health Study, 88% with self-reported active migraine fulfilled either diagnostic criteria of migraine without aura (72%) or probable migraine without aura (16%) [35].

Genotyping in the WGHS was performed with the Illumina Duo “+” platform as described [32], targeting approximately 317K SNPs that tag common variation (i.e. minor allele frequency >∼5%) in populations of European ancestry and supplemented by SNPs to provide dense coverage of candidate genes for cardiovascular disease and related conditions as well as SNPs with known consequences to health. Retained samples had successful genotyping across >98% of the SNPs, while retained SNPs had successful genotyping across >90% of the samples. A multidimensional scaling procedure in PLINK [37] was used to identify the subset of 23,294 WGHS participants with verified self-reported European ancestry. Within this group, SNPs were excluded if a test for Hardy-Weinberg equilibrium had p<10^−6^ or minor allele frequency <1%, leaving 339,596 SNPs in the final data set. Genotypes for additional SNPs in HapMap2 (build 36, r. 22) but not represented on the genotyping array were imputed with MaCH v. 1.0.16 [38] using the CEU reference population.

### Statistical analysis

#### Candidate SNPs

Statistical modeling was applied to the 12 SNPs identified as the lead associations in a recent large genome-wide meta-analysis [13]. Nine of these SNPs had genome-wide significance in meta-analysis incorporating all studies among which there were a total of 23,285 migraine cases. Of the remaining three SNPs, rs10915437 (near *AJAP1*) and rs6790925 (near *TGFBR2*) had genome-wide significance exclusively in clinic-based samples and therefore excluding the WGHS. The final SNP, rs10504861 (near *MMP16*) was discovered exclusively in a sub-analysis including only the 6,550 MO cases, including the 1,826 from the WGHS included in this analysis. In the WGHS, SNPs rs2651899, rs6790925, rs4379368, and rs11172113 were genotyped directly. A small number of missing genotypes from these SNPs and the genotypes at the remaining eight candidate SNPs were imputed. The quality of imputation was adequate or excellent for all of these SNPs ranging from R^2^ values of 0.61 and 0.72 for rs9349379 and rs10915437, respectively, to >0.94 for the remaining SNPs (Table S1).

#### Likelihood framework for model selection

For each of the characteristics accompanying migraine, model selection compared the Bayesian or Akiake Information Criteria (BIC or AIC) penalized likelihood for six different inheritance models. Each inheritance model was specified by SNP minor allele frequencies in three groups: a) migraineurs experiencing the characteristic, b) migraineurs not experiencing the characteristic, and c) non-migraineurs. Following previously published methodology [16], the six possible models were 1) the “null” model, meaning that there was no SNP association with migraine, and specified by the same allele frequency in all three groups (1 degree of freedom [df]); 2) the “basic” model, meaning that the SNP was associated with migraine overall, and specified by one allele frequency among migraineurs regardless of the presence of the characteristic and a different allele frequency among non-migraineurs (2df); 3) the “subset” model, meaning that the SNP was associated with migraine sub-classified according to the presence of one of the characteristics, and specified by one allele frequency among migraineurs experiencing the characteristic and a different but identical minor allele among migraineurs not experiencing the characteristic and non-migraineurs (2df); 4) the “inverse subset” model, meaning that the SNP was associated with migraine sub-classified by the absence of one of the characteristics, and specified by one allele frequency among migraineurs not experiencing the characteristic and a different but identical minor allele among migraineurs experiencing the characteristic and non-migraineurs (2df); 5) the “general” model, meaning that the SNP had different magnitude of association with migraine sub-classified by the presence and the absence of one of the characteristics, and specified by different minor allele frequencies in all three groups (3df); and 6) the “modifier” model, specified by different minor allele frequencies among migraineurs experiencing the characteristic or not experiencing the characteristic, and the weighted mean of the two frequency estimates among non-migraineurs (2df). The “modifier” model describes an association with the migraine characteristic among migraineurs but not between migraineurs and non-migraineurs, i.e. an association with the characteristic conditional on having migraine. The significance of the selected association model for each combination of SNP and migraine associated characteristic was evaluated by the p-value for the standard log-likelihood ratio (LLR) test statistic comparing the likelihood of a selected model with the likelihood of the null model, i.e. assuming the negative of twice the difference in the likelihoods had chi-squared distribution with degrees of freedom equal to twice the difference in the degrees of freedom of the two models under the null.

#### Empirical significance estimates

Empirical significance estimates of the BIC and AIC model selection procedures were derived through a permutation approach. In order to simulate null distributions, the entire BIC and AIC model selection procedures were repeated after random reassignment of SNP genotypes to WGHS participants 10,000 times. For each combination of SNP and migraine characteristic, the probability of selecting a non-null model was estimated as the fraction of non-null models with the permuted data. Similarly, an empirical p-value for the LLR test of the selected model for each combination of SNP and migraine characteristic was estimated as the fraction of LLR test analytic p-values among models from the permutations less than or equal to the observed analytic p-value for a given combination of SNP and migraine characteristic. The permutations were also used to adjust the significance of model LLR tests for multiple hypotheses testing using rank statistics. Thus, for each SNP, a stage 1 correction was made by computing the fraction of permuted results with the smallest LLR test analytic p-value across all 10 migraine characteristics less than or equal to the observed smallest analytic LLR test p-value. Similarly, the second smallest LLR test p-value for that SNP was corrected by computing the fraction of permuted results with second smallest LLR test analytic p-value across all 10 migraine characteristics less than or equal to the observed second smallest LLR test analytic p-value for that SNP. The process was repeated for all 10 LLR test p-values for each SNP. These rank adjusted p-values were then corrected in a second stage using the Šidák procedure assuming 12 independent SNPs.

#### Effect estimates and cluster analysis

SNP effect estimates (beta coefficients) from logistic regression were computed for the association of each SNP with migraine either accompanied or not with each of the characteristics compared with non-migraineurs. The choice of coded allele in the logistic models was assigned as the allele associated with increased probability of any report of migraine in the WGHS population overall at baseline (Table S1). SNPs were clustered according to vectors of the differences in the beta coefficients for migraine with or without each of the characteristics, normalized by the square root of the sum of the squared standard errors, i.e. a t-statistic. The Mahalanobis metric was used to define a distance between each pair of SNP vectors while addressing the potential for correlation structure due to overlap of the migraine characteristics. The covariance of the vector entries for the migraine characteristics in the Mahalanobis analysis was derived from association testing using 1,222 independent SNPs not associated with migraine from the GWAS catalog ([39] available at www.genome.gov/gwastudies, accessed 3/13/2013). Hierarchical clustering was performed with the function “hclust” in R [40].

#### Ethics statement

All data collection and reported research complies with international guidelines and was approved by the Institutional Review Board (IRB) of Brigham and Women's Hospital.

## Supporting Information

Figure S1 Clustering of SNPs according to differential association for migraine characterized by aura or additional features. Scale indicates arbitrary units. See methods for details of clustering procedure.
(DOCX)

Table S1 SNP genotyping and imputation summary.
(DOCX)

Table S2 Empirical significance of BIC selected models derived from permutation analysis. Migraine characteristics designated as in Table 2. See also Methods. A. Fraction BIC models “non-null” from permuted genotypes B. Fraction BIC models from permuted genotypes having LLR test p-value < observed p-value. C. LLR p-values for BIC selected models, corrected for multiple hypothesis testing.
(DOCX)

Table S3 Empirical significance of AIC selected models derived from permutation analysis. Migraine characteristics designated as in Table 2. See also Methods. A. Fraction AIC models “non-null” from permuted genotypes. B. Fraction AIC models from permuted genotypes having LLR test p-value < observed p-value. C. LLR p-values for AIC selected models, corrected for multiple hypothesis testing.
(DOCX)

Table S4 SNP association statistics (beta (SE), p-value) in the WGHS for migraine sub-classified by presence (+) or absence (−) of aura or additional characteristics. Migraine characteristics as in Table 2. Statistics for association with active migraine overall, i.e. without sub-classification, are listed under SNP names in the upper part of the table. SNP encoding as in Table S1.
(DOCX)
