# Correction: Selectivity in Genetic Association with Sub-classified Migraine in Women

**DOI:** 10.1371/journal.pgen.1005330

**Published:** 2015-06-15

**Authors:** Daniel I. Chasman, Verneri Anttila, Julie E. Buring, Paul M. Ridker, Markus Schürks, Tobias Kurth, International Headache Genetics Consortium

Incorrect date for reference [13]:

The date for reference [13] is incorrect. The correct date is 2010, not 2013. The correct reference is: Anttila V, Stefansson H, Kallela M, Todt U, Terwindt GM, et al. (2010) Genome-wide association study of migraine implicates a common susceptibility variant on 8q22.1. Nat Genet 42 (10) 869–873.

Missing reference:

A reference is omitted from the fourth sentence of the second paragraph in the Introduction section.

The sentence should read: Recent reports have described a greater number of highly significant common genetic variants for MO than MA in genome-wide analyses, as well as only partial overlap between the sets of identified genes [11–13, Anttila et al., 2013].

The reference is: Anttila V, Winsvold BS, Gormley P, Kurth T, Bettella F, et al. (2013) Genome-wide meta-analysis identifies new susceptibility loci for migraine. Nat Genet. 45 (8) 912–917.

All subsequent mentions of reference [13] should cite this new reference instead.
