# Supplementary material for: Selectivity in Genetic Association with Sub-classified Migraine in Women
Source: PLoS Genet. 2014 May 22;10(5):e1004366. doi: 10.1371/journal.pgen.1004366 (PMC4031047; doi:10.1371/journal.pgen.1004366)
Supplement: Figure S1 — Clustering of SNPs according to differential association for migraine characterized by aura or additional features. Scale indicates arbitrary units. See methods for details of clustering procedure. (DOCX) [file pgen.1004366.s001.docx]

Figure S1. Clustering of SNPs according to differential association for migraine characterized by aura or additional features. Scale indicates arbitrary units. See methods for details of clustering procedure.
