# Supplementary material for: Selectivity in Genetic Association with Sub-classified Migraine in Women
Source: PLoS Genet. 2014 May 22;10(5):e1004366. doi: 10.1371/journal.pgen.1004366 (PMC4031047; doi:10.1371/journal.pgen.1004366)
Supplement: Table S1 — SNP genotyping and imputation summary. (DOCX) [file pgen.1004366.s002.docx]

| Table S1. SNP genotyping and imputation summary | | | | | | | | |
| --- | --- | --- | --- | --- | --- | --- | --- | --- |
| SNP | chr:pos | candidate gene | A1^ | A2^ | MAF | Rsq* | geno- typed^#^ | coded allele^@^ |
| rs2651899 | 1:3083711 | *PRDM16* | C | T | 0.443 | 0.979 | 1 | C |
| rs10915437 | 1:4183005 | near *AJAP1* | G | A | 0.359 | 0.722 | 0 | G |
| rs12134493 | 1:115677945 | near *TSPAN2* | A | C | 0.119 | 0.984 | 0 | A |
| rs2274316 | 1:156446241 | *MEF2D* | C | A | 0.334 | 0.992 | 0 | C |
| rs7577262 | 2:234818868 | *TRPM8* | A | G | 0.110 | 0.989 | 0 | G |
| rs6790925 | 3:30480084 | near *TGFBR2* | T | C | 0.358 | 0.998 | 1 | T |
| rs9349379 | 6:12903956 | *PHACTR1* | G | A | 0.377 | 0.615 | 0 | A |
| rs13208321 | 6:96860353 | *FHL5* | A | G | 0.224 | 0.995 | 0 | A |
| rs4379368 | 7:40466199 | C7orf10 | T | C | 0.102 | 0.999 | 1 | T |
| rs10504861 | 8:89547931 | near *MMP16* | T | C | 0.180 | 0.964 | 0 | C |
| rs6478241 | 9:119252628 | *ASTN2* | A | G | 0.363 | 0.934 | 0 | A |
| rs11172113 | 12:57527282 | *LRP1* | C | T | 0.409 | 0.993 | 1 | T |
| ^A1=minor allele, A2=major allele  *Imputation quality Rsq from MaCH v.1.0.16 | | | | | | | | |
| ^#^Imputation at genotyped SNPs was used to complete missing data, max. missing 0.7%. | | | | | | | | |
| ^@^coded allele for Figure 1 and Table S4 | | | | | | | | |
