# Supplementary material for: Selectivity in Genetic Association with Sub-classified Migraine in Women
Source: PLoS Genet. 2014 May 22;10(5):e1004366. doi: 10.1371/journal.pgen.1004366 (PMC4031047; doi:10.1371/journal.pgen.1004366)
Supplement: Table S2 — Empirical significance of BIC selected models derived from permutation analysis. Migraine characteristics designated as in Table 2. See also Methods. A. Fraction BIC models “non-null” from permuted genotypes B. Fraction BIC models from permuted genotypes having LLR test p-value < observed p-value. C. LLR p-values for BIC selected models, corrected for multiple hypothesis testing. (DOCX) [file pgen.1004366.s003.docx]

Table S2. Empirical significance of BIC selected models derived from permutation analysis. Migraine characteristics designated as in Table 2.

A. Fraction BIC models “non-null” from permuted genotypes

| SNP | aura | pulsate | unipain | sound | light | longdur | nausea | aggrphys | inhibit | freq |
| --- | --- | --- | --- | --- | --- | --- | --- | --- | --- | --- |
| rs2651899 | 0.0032 | 0.0044 | 0.0032 | 0.0045 | 0.0038 | 0.0043 | 0.0049 | 0.0047 | 0.0039 | 0.0042 |
| rs10915437 | 0.0010 | 0.0006 | 0.0004 | 0.0006 | 0.0002 | 0.0003 | 0.0004 | 0.0005 | 0.0010 | 0.0007 |
| rs12134493 | 0.0031 | 0.0043 | 0.0044 | 0.0042 | 0.0040 | 0.0031 | 0.0042 | 0.0052 | 0.0053 | 0.0041 |
| rs2274316 | 0.0044 | 0.0054 | 0.0051 | 0.0049 | 0.0046 | 0.0047 | 0.0054 | 0.0046 | 0.0055 | 0.0050 |
| rs7577262 | 0.0044 | 0.0035 | 0.0047 | 0.0032 | 0.0045 | 0.0040 | 0.0039 | 0.0035 | 0.0050 | 0.0032 |
| rs6790925 | 0.0047 | 0.0053 | 0.0039 | 0.0046 | 0.0039 | 0.0045 | 0.0045 | 0.0046 | 0.0042 | 0.0032 |
| rs9349379 | 0.0003 | 0.0002 | 0.0002 | 0.0003 | 0.0004 | 0.0002 | 0.0002 | 0.0002 | 0.0003 | 0.0002 |
| rs13208321 | 0.0056 | 0.0049 | 0.0066 | 0.0046 | 0.0044 | 0.0045 | 0.0038 | 0.0048 | 0.0046 | 0.0049 |
| rs4379368 | 0.0040 | 0.0045 | 0.0038 | 0.0051 | 0.0036 | 0.0041 | 0.0038 | 0.0048 | 0.0037 | 0.0048 |
| rs10504861 | 0.0041 | 0.0034 | 0.0035 | 0.0038 | 0.0036 | 0.0038 | 0.0036 | 0.0042 | 0.0038 | 0.0035 |
| rs6478241 | 0.0029 | 0.0032 | 0.0042 | 0.0034 | 0.0039 | 0.0031 | 0.0037 | 0.0044 | 0.0037 | 0.0034 |
| rs11172113 | 0.0048 | 0.0045 | 0.0052 | 0.0050 | 0.0052 | 0.0037 | 0.0050 | 0.0045 | 0.0043 | 0.0048 |

B. Fraction BIC models from permuted genotypes having LLR test p-value < observed p-value

| SNP | aura | pulsate | unipain | sound | light | longdur | nausea | aggrphys | inhibit | freq |
| --- | --- | --- | --- | --- | --- | --- | --- | --- | --- | --- |
| rs2651899 | 0.0002 | 0.0002 | 0.0004 | 0.0005 | <0.0001 | 0.0001 | <0.0001 | <0.0001 | <0.0001 | 0.0002 |
| rs10915437 | 1 | 1 | 1 | 1 | 1 | 1 | 1 | 1 | 1 | 1 |
| rs12134493 | 0.0004 | 0.0002 | 0.0002 | 0.0001 | 0.0002 | 0.0003 | 0.0002 | 0.0001 | 0.0002 | 0.0002 |
| rs2274316 | 1 | 1 | 1 | 1 | 1 | 1 | 1 | 1 | 1 | 1 |
| rs7577262 | 0.0003 | <0.0001 | 0.0002 | 0.0002 | <0.0001 | 0.0001 | 0.0003 | 0.0001 | 0.0002 | 0.0004 |
| rs6790925 | 1 | 1 | 1 | 1 | 1 | 1 | 1 | 1 | 1 | 1 |
| rs9349379 | 1 | 1 | 1 | 1 | 1 | 1 | 1 | 1 | 1 | 1 |
| rs13208321 | 1 | 1 | 1 | 1 | 1 | 1 | 0.0022 | 1 | 1 | 1 |
| rs4379368 | 1 | 1 | 1 | 1 | 1 | 1 | 1 | 1 | 1 | 1 |
| rs10504861 | 1 | 0.0006 | 0.0032 | 0.0026 | 0.0021 | 0.0030 | 0.0006 | 1 | 1 | 1 |
| rs6478241 | 1 | 1 | 1 | 1 | 1 | 1 | 1 | 1 | 1 | 1 |
| rs11172113 | 0.0002 | 0.0003 | 0.0002 | 0.0006 | 0.0008 | 0.0006 | 0.0009 | 0.0001 | 0.0005 | 0.0005 |

C. LLR p-values for BIC selected models, corrected for multiple hypothesis testing

| SNP | aura | pulsate | unipain | sound | light | longdur | nausea | aggrphys | inhibit | freq |
| --- | --- | --- | --- | --- | --- | --- | --- | --- | --- | --- |
| rs2651899 | <0.001 | <0.001 | <0.001 | <0.001 | <0.001 | <0.001 | <0.001 | <0.001 | <0.001 | <0.001 |
| rs10915437 | 1 | 1 | 1 | 1 | 1 | 1 | 1 | 1 | 1 | 1 |
| rs12134493 | 0.001 | 0.001 | 0.001 | 0.001 | 0.001 | 0.001 | 0.001 | 0.001 | 0.001 | 0.002 |
| rs2274316 | 1 | 1 | 1 | 1 | 1 | 1 | 1 | 1 | 1 | 1 |
| rs7577262 | 0.021 | <0.001 | <0.001 | <0.001 | <0.001 | <0.001 | <0.001 | <0.001 | <0.001 | <0.001 |
| rs6790925 | 1 | 1 | 1 | 1 | 1 | 1 | 1 | 1 | 1 | 1 |
| rs9349379 | 1 | 1 | 1 | 1 | 1 | 1 | 1 | 1 | 1 | 1 |
| rs13208321 | 1 | 1 | 1 | 1 | 1 | 1 | 0.188 | 1 | 1 | 1 |
| rs4379368 | 1 | 1 | 1 | 1 | 1 | 1 | 1 | 1 | 1 | 1 |
| rs10504861 | 1 | 0.069 | 0.012 | 0.011 | 0.013 | 0.012 | 0.006 | 1 | 1 | 1 |
| rs6478241 | 1 | 1 | 1 | 1 | 1 | 1 | 1 | 1 | 1 | 1 |
| rs11172113 | 0.028 | 0.002 | 0.001 | 0.001 | 0.001 | 0.004 | 0.001 | 0.001 | 0.001 | 0.001 |
