# Supplementary material for: Selectivity in Genetic Association with Sub-classified Migraine in Women
Source: PLoS Genet. 2014 May 22;10(5):e1004366. doi: 10.1371/journal.pgen.1004366 (PMC4031047; doi:10.1371/journal.pgen.1004366)
Supplement: Table S3 — Empirical significance of AIC selected models derived from permutation analysis. Migraine characteristics designated as in Table 2. See also Methods. A. Fraction AIC models “non-null” from permuted genotypes. B. Fraction AIC models from permuted genotypes having LLR test p-value < observed p-value. C. LLR p-values for AIC selected models, corrected for multiple hypothesis testing. (DOCX) [file pgen.1004366.s004.docx]

Table S3. Empirical significance of AIC selected models. Migraine characteristics designated as in Table 2.

A. Fraction AIC models “non-null” from permuted genotypes

| SNP | aura | pulsate | Unipain | sound | light | longdur | nausea | aggrphys | inhibit | freq |
| --- | --- | --- | --- | --- | --- | --- | --- | --- | --- | --- |
| rs2651899 | 0.33 | 0.34 | 0.33 | 0.34 | 0.34 | 0.32 | 0.34 | 0.33 | 0.35 | 0.33 |
| rs10915437 | 0.23 | 0.23 | 0.23 | 0.23 | 0.23 | 0.22 | 0.22 | 0.23 | 0.23 | 0.23 |
| rs12134493 | 0.32 | 0.32 | 0.32 | 0.32 | 0.32 | 0.32 | 0.32 | 0.32 | 0.33 | 0.32 |
| rs2274316 | 0.33 | 0.34 | 0.33 | 0.33 | 0.33 | 0.32 | 0.33 | 0.33 | 0.34 | 0.33 |
| rs7577262 | 0.32 | 0.33 | 0.33 | 0.33 | 0.33 | 0.32 | 0.33 | 0.33 | 0.33 | 0.33 |
| rs6790925 | 0.34 | 0.34 | 0.34 | 0.34 | 0.33 | 0.33 | 0.33 | 0.33 | 0.34 | 0.34 |
| rs9349379 | 0.17 | 0.17 | 0.17 | 0.17 | 0.17 | 0.16 | 0.17 | 0.17 | 0.17 | 0.17 |
| rs13208321 | 0.34 | 0.35 | 0.34 | 0.34 | 0.34 | 0.33 | 0.34 | 0.34 | 0.34 | 0.34 |
| rs4379368 | 0.34 | 0.35 | 0.35 | 0.34 | 0.34 | 0.33 | 0.34 | 0.35 | 0.35 | 0.34 |
| rs10504861 | 0.33 | 0.33 | 0.33 | 0.32 | 0.32 | 0.32 | 0.32 | 0.32 | 0.33 | 0.33 |
| rs6478241 | 0.32 | 0.31 | 0.32 | 0.31 | 0.32 | 0.31 | 0.32 | 0.32 | 0.32 | 0.32 |
| rs11172113 | 0.33 | 0.34 | 0.33 | 0.34 | 0.33 | 0.33 | 0.33 | 0.33 | 0.33 | 0.33 |

B. Fraction AIC models from permuted genotypes having LLR test p-value < observed p-value

| SNP | aura | pulsate | unipain | sound | light | longdur | nausea | aggrphys | inhibit | freq |
| --- | --- | --- | --- | --- | --- | --- | --- | --- | --- | --- |
| rs2651899 | 0.0002 | 0.0002 | 0.0004 | 0.0005 | <0.0001 | 0.0001 | <0.0001 | <0.0001 | <0.0001 | 0.0002 |
| rs10915437 | 0.0598 | 0.1900 | 1 | 1 | 1 | 1 | 1 | 1 | 1 | 1 |
| rs12134493 | 0.0004 | 0.0002 | 0.0002 | 0.0001 | 0.0002 | 0.0003 | 0.0002 | 0.0001 | 0.0002 | 0.0002 |
| rs2274316 | 1 | 0.0372 | 1 | 1 | 1 | 1 | 1 | 1 | 0.0657 | 0.2351 |
| rs7577262 | 0.0003 | <0.0001 | 0.0002 | 0.0002 | <0.0001 | 0.0001 | 0.0003 | 0.0001 | 0.0002 | 0.0004 |
| rs6790925 | 1 | 1 | 0.2837 | 0.0109 | 0.0426 | 0.3264 | 0.0123 | 0.1788 | 0.0903 | 0.1431 |
| rs9349379 | 0.0007 | 0.0390 | 0.0456 | 0.0408 | 0.0392 | 0.0397 | 0.0402 | 0.0266 | 0.0413 | 0.0380 |
| rs13208321 | 0.0178 | 0.0334 | 0.0330 | 0.0324 | 0.0349 | 0.0082 | 0.0022 | 0.0116 | 0.0070 | 0.0340 |
| rs4379368 | 0.1711 | 0.0743 | 0.1714 | 0.1737 | 0.1638 | 0.1654 | 0.1741 | 0.1562 | 0.0333 | 0.1301 |
| rs10504861 | 0.0175 | 0.0006 | 0.0033 | 0.0026 | 0.0023 | 0.0030 | 0.0008 | 0.0176 | 0.0172 | 0.0171 |
| rs6478241 | 0.0158 | 0.0462 | 0.0412 | 0.0440 | 0.0300 | 0.0435 | 0.0481 | 0.0466 | 0.0452 | 0.0465 |
| rs11172113 | 0.0002 | 0.0003 | 0.0002 | 0.0004 | 0.0008 | 0.0006 | 0.0008 | 0.0001 | 0.0005 | 0.0005 |

C. LLR p-values for AIC selected models, corrected for multiple hypothesis testing

| SNP | aura | pulsate | unipain | sound | light | longdur | nausea | aggrphys | inhibit | freq |
| --- | --- | --- | --- | --- | --- | --- | --- | --- | --- | --- |
| rs2651899 | <0.001 | <0.001 | <0.001 | <0.001 | <0.001 | <0.001 | <0.001 | <0.001 | <0.001 | <0.001 |
| rs10915437 | 0.978 | 0.995 | 1 | 1 | 1 | 1 | 1 | 1 | 1 | 1 |
| rs12134493 | 0.001 | 0.001 | 0.001 | 0.001 | 0.001 | 0.001 | 0.001 | 0.001 | 0.001 | 0.004 |
| rs2274316 | 1 | 0.905 | 1 | 1 | 1 | 1 | 1 | 1 | 0.756 | 0.984 |
| rs7577262 | 0.021 | <0.001 | <0.001 | <0.001 | <0.001 | <0.001 | <0.001 | <0.001 | <0.001 | <0.001 |
| rs6790925 | 1 | 1 | 0.821 | 0.513 | 0.345 | 0.880 | 0.198 | 0.668 | 0.506 | 0.614 |
| rs9349379 | 0.065 | 0.329 | 0.245 | 0.201 | 0.178 | 0.171 | 0.170 | 0.389 | 0.170 | 0.170 |
| rs13208321 | 0.082 | 0.146 | 0.143 | 0.140 | 0.139 | 0.055 | 0.187 | 0.055 | 0.100 | 0.138 |
| rs4379368 | 0.711 | 0.805 | 0.637 | 0.597 | 0.611 | 0.603 | 0.595 | 0.769 | 0.885 | 0.838 |
| rs10504861 | 0.071 | 0.071 | 0.012 | 0.011 | 0.013 | 0.012 | 0.007 | 0.070 | 0.070 | 0.070 |
| rs6478241 | 0.665 | 0.214 | 0.337 | 0.202 | 0.417 | 0.193 | 0.189 | 0.189 | 0.246 | 0.189 |
| rs11172113 | 0.028 | 0.001 | 0.001 | 0.001 | 0.001 | 0.004 | 0.001 | 0.001 | 0.001 | 0.001 |
