# Supplementary material for: Selectivity in Genetic Association with Sub-classified Migraine in Women
Source: PLoS Genet. 2014 May 22;10(5):e1004366. doi: 10.1371/journal.pgen.1004366 (PMC4031047; doi:10.1371/journal.pgen.1004366)
Supplement: Table S4 — SNP association statistics (beta (SE), p-value) in the WGHS for migraine sub-classified by presence (+) or absence (−) of aura or additional characteristics. Migraine characteristics as in Table 2. Statistics for association with active migraine overall, i.e. without sub-classification, are listed under SNP names in the upper part of the table. SNP encoding as in Table S1. (DOCX) [file pgen.1004366.s005.docx]

Table S4. SNP association statistics (beta (SE), p-value) in the WGHS for migraine sub-classified by presence (+) or absence (-) of additional characteristics. Migraine characteristics as in Table 2. Active migraine association statistics are listed under SNP names in the upper part of the table. SNP encoding as in Table S1.

| SNP |  | migraine characteristic | | | | |
| --- | --- | --- | --- | --- | --- | --- |
|  |  | aura | pulsating pain | unilateral pain | phonophobia | photophobia |
| rs7577262 | + | 0.11 (0.070), 0.11 | 0.14 (0.061), 0.027 | 0.16 (0.059), 0.0051 | 0.13 (0.069), 0.068 | 0.15 (0.056), 0.0079 |
| 0.181 (0.047), 1.2E-04 | - | 0.23 (0.060), 0.00014 | 0.23 (0.067), 0.00057 | 0.20 (0.072), 0.0043 | 0.22 (0.060), 0.00026 | 0.24 (0.079), 0.0019 |
| rs11172113 | + | 0.057 (0.043), 0.19 | 0.11 (0.038), 0.0031 | 0.11 (0.036), 0.003 | 0.071 (0.043), 0.097 | 0.10 (0.034), 0.0038 |
| 0.108 (0.029), 1.6E-04 | - | 0.14 (0.036), 8.3e-05 | 0.10 (0.040), 0.01 | 0.11 (0.043), 0.011 | 0.13 (0.036), 0.00023 | 0.12 (0.047), 0.0081 |
| rs6478241 | + | 0.019 (0.045), 0.67 | 0.079 (0.039), 0.043 | 0.092 (0.037), 0.013 | 0.079 (0.044), 0.072 | 0.092 (0.035), 0.0093 |
| 0.071 (0.030), 0.017 | - | 0.10 (0.037), 0.0048 | 0.061 (0.041), 0.14 | 0.038 (0.045), 0.39 | 0.065 (0.037), 0.083 | 0.029 (0.048), 0.55 |
| rs10915437 | + | -0.06 (0.052), 0.25 | 0.077 (0.045), 0.084 | 0.019 (0.043), 0.65 | 0.048 (0.050), 0.34 | 0.035 (0.041), 0.4 |
| 0.033 (0.034), 0.327 | - | 0.092 (0.042), 0.028 | -0.017 (0.048), 0.72 | 0.054 (0.051), 0.29 | 0.023 (0.043), 0.59 | 0.031 (0.055), 0.58 |
| rs9349379 | + | -0.026 (0.056), 0.64 | 0.12 (0.049), 0.018 | 0.083 (0.047), 0.075 | 0.12 (0.056), 0.029 | 0.067 (0.044), 0.13 |
| 0.091 (0.037), 0.014 | - | 0.17 (0.047), 0.00031 | 0.063 (0.052), 0.23 | 0.10 (0.056), 0.065 | 0.07 (0.047), 0.14 | 0.14 (0.061), 0.024 |
| rs12134493 | + | 0.13 (0.064), 0.046 | 0.19 (0.054), 0.00037 | 0.22 (0.051), 1.7e-05 | 0.26 (0.060), 1.6e-05 | 0.20 (0.049), 3.3e-05 |
| 0.167 (0.041), 5.5E-05 | - | 0.19 (0.051), 0.00017 | 0.14 (0.058), 0.019 | 0.086 (0.064), 0.18 | 0.10 (0.053), 0.06 | 0.093 (0.069), 0.17 |
| rs6790925 | + | -0.01 (0.044), 0.82 | 0.036 (0.038), 0.35 | 0.054 (0.036), 0.13 | 0.12 (0.043), 0.0049 | 0.077 (0.035), 0.026 |
| 0.024 (0.029), 0.402 | - | 0.046 (0.036), 0.2 | 0.011 (0.041), 0.78 | -0.02 (0.044), 0.64 | -0.044 (0.037), 0.23 | -0.08 (0.048), 0.096 |
| rs13208321 | + | 0.035 (0.051), 0.48 | 0.11 (0.043), 0.0094 | 0.11 (0.041), 0.0096 | 0.12 (0.048), 0.011 | 0.09 (0.039), 0.022 |
| 0.084 (0.033), 0.011 | - | 0.11 (0.041), 0.0049 | 0.051 (0.046), 0.27 | 0.05 (0.050), 0.31 | 0.056 (0.042), 0.18 | 0.071 (0.054), 0.19 |
| rs10504861 | + | 0.059 (0.057), 0.3 | 0.18 (0.052), 0.00036 | 0.16 (0.048), 0.0013 | 0.20 (0.058), 0.00083 | 0.16 (0.046), 0.00081 |
| 0.104 (0.038), 0.006 | - | 0.13 (0.048), 0.005 | 0.018 (0.052), 0.72 | 0.03 (0.056), 0.59 | 0.043 (0.047), 0.36 | 0.0095 (0.060), 0.87 |
| rs2651899 | + | 0.12 (0.043), 0.0037 | 0.12 (0.037), 0.0014 | 0.12 (0.035), 0.00078 | 0.16 (0.042), 0.00014 | 0.14 (0.034), 1.9e-05 |
| 0.110 (0.028), 9.4E-05 | - | 0.10 (0.035), 0.0042 | 0.099 (0.039), 0.012 | 0.097 (0.042), 0.023 | 0.075 (0.036), 0.035 | 0.043 (0.046), 0.35 |
| rs2274316 | + | 0.032 (0.045), 0.48 | 0.094 (0.039), 0.016 | 0.045 (0.037), 0.23 | 0.043 (0.044), 0.33 | 0.034 (0.036), 0.33 |
| 0.021 (0.030), 0.472 | - | 0.014 (0.037), 0.7 | -0.062 (0.042), 0.14 | -0.013 (0.045), 0.76 | 0.0063 (0.038), 0.87 | -0.004 (0.048), 0.93 |
| rs4379368 | + | 0.051 (0.070), 0.46 | 0.13 (0.059), 0.023 | 0.061 (0.057), 0.29 | 0.12 (0.067), 0.077 | 0.074 (0.055), 0.17 |
| 0.083 (0.045), 0.067 | - | 0.10 (0.056), 0.065 | 0.023 (0.065), 0.72 | 0.12 (0.067), 0.086 | 0.058 (0.058), 0.31 | 0.10 (0.073), 0.17 |
| SNP |  | migraine characteristic | | | | |
|  |  | duration of 4-72 hours | nausea | aggr. phys. act. | inhibits daily act. | ≥6 attacks/year |
| rs7577262 | + | 0.17 (0.052), 0.0014 | 0.21 (0.057), 3e-04 | 0.11 (0.075), 0.13 | 0.18 (0.064), 0.0055 | 0.20 (0.077), 0.0084 |
|  | - | 0.23 (0.097), 0.016 | 0.13 (0.075), 0.078 | 0.22 (0.057), 0.00015 | 0.18 (0.064), 0.0044 | 0.17 (0.057), 0.0028 |
| rs11172113 | + | 0.12 (0.032), 0.00012 | 0.12 (0.035), 0.00053 | 0.13 (0.047), 0.0048 | 0.12 (0.039), 0.0028 | 0.13 (0.046), 0.0037 |
|  | - | 0.054 (0.058), 0.34 | 0.085 (0.046), 0.064 | 0.095 (0.034), 0.0054 | 0.099 (0.039), 0.011 | 0.094 (0.035), 0.0067 |
| rs6478241 | + | 0.077 (0.033), 0.019 | 0.073 (0.036), 0.042 | 0.044 (0.048), 0.36 | 0.10 (0.040), 0.013 | 0.11 (0.047), 0.022 |
|  | - | 0.047 (0.060), 0.43 | 0.067 (0.048), 0.16 | 0.084 (0.035), 0.017 | 0.041 (0.040), 0.31 | 0.05 (0.036), 0.16 |
| rs10915437 | + | 0.029 (0.038), 0.45 | 0.052 (0.041), 0.2 | 0.0099 (0.055), 0.86 | 0.027 (0.046), 0.56 | 0.079 (0.054), 0.14 |
|  | - | 0.049 (0.068), 0.47 | -0.0027 (0.055), 0.96 | 0.045 (0.041), 0.27 | 0.04 (0.046), 0.39 | 0.0083 (0.041), 0.84 |
| rs9349379 | + | 0.092 (0.041), 0.026 | 0.082 (0.045), 0.067 | 0.039 (0.060), 0.52 | 0.087 (0.051), 0.085 | 0.14 (0.060), 0.018 |
|  | - | 0.089 (0.075), 0.24 | 0.11 (0.060), 0.072 | 0.12 (0.045), 0.0081 | 0.095 (0.050), 0.061 | 0.064 (0.045), 0.16 |
| rs12134493 | + | 0.18 (0.046), 0.00014 | 0.18 (0.050), 0.00025 | 0.20 (0.067), 0.0029 | 0.17 (0.056), 0.0026 | 0.31 (0.063), 1e-06 |
|  | - | 0.14 (0.084), 0.1 | 0.14 (0.067), 0.038 | 0.15 (0.050), 0.0025 | 0.16 (0.056), 0.0034 | 0.084 (0.051), 0.1 |
| rs6790925 | + | 0.044 (0.032), 0.17 | 0.086 (0.035), 0.013 | 0.083 (0.047), 0.08 | 0.08 (0.039), 0.041 | 0.087 (0.046), 0.062 |
|  | - | -0.047 (0.059), 0.42 | -0.095 (0.048), 0.047 | -0.0058 (0.035), 0.87 | -0.032 (0.040), 0.42 | -0.0096 (0.035), 0.78 |
| rs13208321 | + | 0.11 (0.036), 0.0028 | 0.13 (0.039), 0.00086 | 0.16 (0.053), 0.0027 | 0.14 (0.044), 0.0019 | 0.12 (0.052), 0.018 |
|  | - | -0.0081 (0.068), 0.91 | -0.0073 (0.054), 0.89 | 0.045 (0.040), 0.26 | 0.029 (0.045), 0.52 | 0.062 (0.040), 0.12 |
| rs10504861 | + | 0.14 (0.043), 0.0012 | 0.16 (0.047), 0.00043 | 0.17 (0.064), 0.0065 | 0.14 (0.052), 0.0087 | 0.14 (0.062), 0.025 |
|  | - | -0.013 (0.074), 0.86 | -0.0032 (0.059), 0.96 | 0.07 (0.045), 0.12 | 0.071 (0.051), 0.16 | 0.085 (0.046), 0.061 |
| rs2651899 | + | 0.11 (0.031), 0.00027 | 0.16 (0.034), 4.8e-06 | 0.22 (0.046), 2.4e-06 | 0.18 (0.038), 1.5e-06 | 0.086 (0.045), 0.058 |
|  | - | 0.094 (0.057), 0.096 | 0.025 (0.046), 0.59 | 0.055 (0.034), 0.1 | 0.035 (0.038), 0.36 | 0.12 (0.034), 3e-04 |
| rs2274316 | + | 0.027 (0.033), 0.4 | 0.026 (0.036), 0.47 | 0.06 (0.048), 0.21 | 0.086 (0.040), 0.032 | 0.076 (0.047), 0.11 |
|  | - | -8e-04 (0.060), 0.99 | 0.013 (0.048), 0.79 | 0.0011 (0.036), 0.98 | -0.044 (0.041), 0.28 | -0.0085 (0.036), 0.81 |
| rs4379368 | + | 0.078 (0.050), 0.12 | 0.096 (0.054), 0.076 | 0.14 (0.072), 0.05 | 0.15 (0.060), 0.01 | 0.15 (0.071), 0.041 |
|  | - | 0.10 (0.090), 0.27 | 0.058 (0.074), 0.43 | 0.052 (0.055), 0.34 | 0.0075 (0.063), 0.91 | 0.048 (0.055), 0.38 |
